# Supplementary material for: Local and global density have distinct and parasite-dependent effects on infection in wild sheep
Source: Parasitology. 2025 Jul 1;152(7):715–23. doi: 10.1017/S0031182025100383 (PMC12418280; doi:10.1017/S0031182025100383)
Supplement: Albery et al. supplementary material 1 — Albery et al. supplementary material [file S0031182025100383sup001.docx]

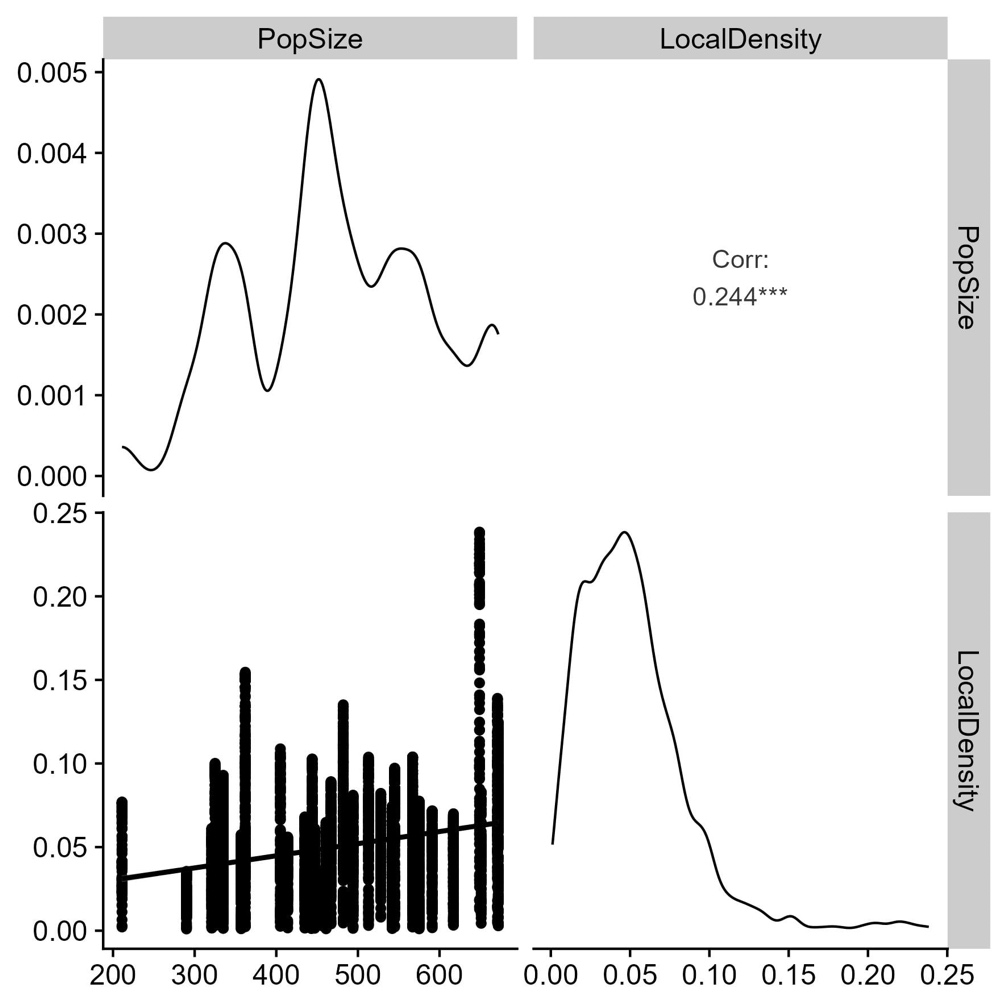


Supplementary Figure 1. Pairwise local and global density (=population size) correlations for our full dataset. The correlation value represents a Pearson correlation, significant at P<0.001.
